# *De novo* transcriptome assembly and analysis of differentially expressed genes of two barley genotypes reveal root-zone-specific responses to salt exposure

**DOI:** 10.1038/srep31558

**Published:** 2016-08-16

**Authors:** Camilla Beate Hill, Andrew Cassin, Gabriel Keeble-Gagnère, Monika S. Doblin, Antony Bacic, Ute Roessner

**Affiliations:** 1 School of BioSciences, The University of Melbourne, Parkville, Vic 3010, Australia; 2 ARC Centre of Excellence in Plant Cell Walls, School of BioSciences, The University of Melbourne, Parkville, Vic 3010, Australia; 3 School of Veterinary and Life Sciences, Murdoch University, Murdoch, WA 6150, Australia

## Abstract

Plant roots are the first organs sensing and responding to salinity stress, manifested differentially between different root types, and also at the individual tissue and cellular level. High genetic diversity and the current lack of an assembled map-based sequence of the barley genome severely limit barley research potential. We used over 580 and 600 million paired-end reads, respectively, to create two *de novo* assemblies of a barley landrace (Sahara) and a malting cultivar (Clipper) with known contrasting responses to salinity. Generalized linear models were used to statistically access spatial, treatment-related, and genotype-specific responses. This revealed a spatial gene expression gradient along the barley root, with more differentially expressed transcripts detected between different root zones than between treatments. The root transcriptome also showed a gradual transition from transcripts related to sugar-mediated signaling at the root meristematic zone to those involved in cell wall metabolism in the elongation zone, and defense response-related pathways toward the maturation zone, with significant differences between the two genotypes. The availability of these additional transcriptome reference sets will serve as a valuable resource to the cereal research community, and may identify valuable traits to assist in breeding programmes.

Barley (*Hordeum vulgare* L.) is an essential food, feed and brewing crop, and a model system for temperate cereals. As a glycophyte, barley suffers substantial yield loss when grown under saline conditions^1^. Plant roots are the first organs sensing and responding to environmental stresses, including salinity stress, and have key functions in water and nutrient uptake and rhizosphere dynamics as well as anchoring the plant^2^. These responses can be manifested differentially between different root types, and also at the individual tissue and cellular level, as the longitudinal structure of plant roots contains partially overlapping specialized zones of development: The plant root tip includes the root cap and the apical meristem, where cell division and elongation originate and proceed along a developmental gradient toward the mature root^3^,^4^. The elongation zone is where newly generated cells increase in length, and can be further sub-divided into the distal elongation zone, a transition zone between meristematic and elongation zone. The distal elongation zone is bordered by the elongation zone, where cells cease division but elongate maximally, and which is adjacent to the maturation zone, where cells can further differentiate into specialized cell types, such as root hairs.

Genome-wide expression profiles of mRNA under both control and stress conditions have revealed striking cell-type and tissue-specific responses in plant roots^5^,^6^,^7^. These studies show that the analysis of spatially (and temporally) resolved transcriptional signatures along longitudinal root sections can be used to infer root developmental processes, and to predict previously unknown cellular functions through co-expression with genes of known function. This strongly suggests that analyses of whole plant tissues can dilute out information important to understand the complex molecular programmes that define root development^6^ and responses to stress^8^.

Relatively little is understood of barley roots during early developmental stages and under exposure to salinity stress. This is due in part to the lack of comprehensive barley root sequence datasets that limits the scope of investigations into the molecular and genetic basis of root traits. The assembly of genome sequences for barley cultivars “Morex”, “Bowman”, and “Barke”, was completed recently. However, the extensive genetic diversity (estimated 370,796 accessions of 31 *Hordeum* species^9^) and the current lack of an assembled map-based barley reference genome sequence still limit research potential. Given the substantial divergence among cultivars and ecotypes, *de novo* transcriptome assemblies should not be limited to species without published reference genomes: recent studies have shown that de novo transcriptome assemblies of plants with sequenced genomes can improve the annotation of diverse cultivars and identify cultivar-specific genes^10^. The comparison of polymorphisms against a single reference genotype does not necessarily provide a complete representation of the genetic diversity of a species, and may underestimate the variability among different genotypes. To discover previously unrecognized transcripts not part of the reference genome as well as to capture potentially novel transcript diversities between the different barley genotypes we have performed two separate *de novo* assemblies, one for the barley malting cultivar (cv.) Clipper (Australia), and one for the landrace (LR) Sahara (North Africa).

Clipper and Sahara are of particular interest due to their contrasting salt tolerance^11^,^12^,^13^. Clipper contains the Na^+^ exclusion locus *HvNax4* which was shown to reduce shoot Na^+^ content by 12–59% (g^−1^ dry weight)^14^. Shelden *et al*.^15^ reported altered root growth phenotypes between Clipper and Sahara in response to the early phase of salinity, with Clipper able to maintain a significantly higher relative root elongation rate (RRER) than Sahara.

The key aims of this study were: (a) to provide new comprehensive resources for the identification of novel potential candidate genes for salinity tolerance in barley; and (b) to quantify the changes in transcript expression under varying conditions in functionally different zones of the root. We achieved this by constructing two independent *de novo* transcriptome assemblies of three key zones of barley roots (meristematic zone, elongation zone, and maturation zone) of an Australian malting cultivar and a North African landrace, before and after salinity stress, based on next generation RNA sequencing (RNA-Seq) to uncover the expressed gene complement. Comparative transcriptome profiling provided insights into the molecular and physiological functions in barley roots, and spatially resolved transcriptional information to reveal both unique genotype-specific as well as treatment-specific features. These reference transcriptomes provide resources for further molecular investigations of the *Hordeum* genus to reveal novel processes of root growth and development under salinity stress, and to identify candidate genes that will inform future crop improvement programs.

## Results

### High quality *de novo* transcriptome assemblies of root longitudinal sections obtained from two barley genotypes

Paired-end RNA-Seq libraries were constructed for three root sections of the meristematic zone (including root tip; Z1), elongation zone (Z2), and maturation zone (Z3) of the roots of barley cv. Clipper, and LR Sahara, grown under control and salt-treated (100 mM NaCl) conditions in quadruplicate. Each sample, regardless of genotype, treatment or biological replicate, was represented by at least 32 million reads, after trimming and confirmation of a tag density sufficient for quantitative analysis of gene expression. The sequence reads were assembled using the Trinity assembler^16^,^17^, and the *de novo* transcriptome assembly summary statistics are summarised in Table 1.

We aligned quality-trimmed reads of each sample independently back to the transcriptome to identify per-sample variability. For Clipper samples, a mean of 10.2% of reads did not align to the Clipper assembled transcriptome (SD 0.4), 40.9% of reads aligned in a single concordant location (SD 1.9), and 48.9% (SD 1.7) concordantly aligned to more than one location in the transcriptome. For Sahara, 9.2% of reads did not align to the Sahara assembled transcriptome (SD 0.4), a per-sample average of 38.9% aligned concordantly exactly once (SD 1.6), whilst 51.9% of paired-reads aligned to more than one contig in the Sahara transcriptome (SD 1.3).

### Validation of genotype assemblies

To validate each assembly, we pursued four separate approaches: (i) validation of core eukaryotic genes, (ii) reciprocal cross-validation of each genotype, (iii) comparative genomics analysis using available public plant genome assemblies from Phytozome (http://www.phytozome.net, version 10.2) and (iv) validation of the presence of key salt-responsive genes.

### Presence of widely conserved eukaryotic genes

We used the Core Eukaryotic Genes Mapping Approach (CEGMA) program v2.4.010312^18^ to detect the presence of 248 widely conserved eukaryotic core genes (CEG) that are considered to have low frequencies of gene family expansion. For the Clipper assembly, we found that 242 of 248 CEGMA genes are complete in our gene list (97.6%), and an additional 6 genes are represented as fragments, with a total representation of 100% of the eukaryotic core gene set (Supplemental Table S1). For the Sahara assembly, we found that 240 of 248 CEGMA genes are complete (96.8%) in our gene list, and an additional 5 genes are represented as fragments, with a total representation of 98.8% of the eukaryotic core gene set (Supplemental Table S2). In comparison, 95% and 95.6% of the complete CEGs were previously identified in tobacco (*Nicotiana benthamiana*)^19^ and alfalfa (*Medicago sativa* L.) transcriptomes^20^, respectively. Using completeness and presence of CEGs as metrics of transcriptome assembly success, this suggests we have generated two high-quality transcriptome assemblies of Clipper and Sahara.

### Reciprocal cross-validation shows high sequence similarity between barley genotypes

**Figure 1:**
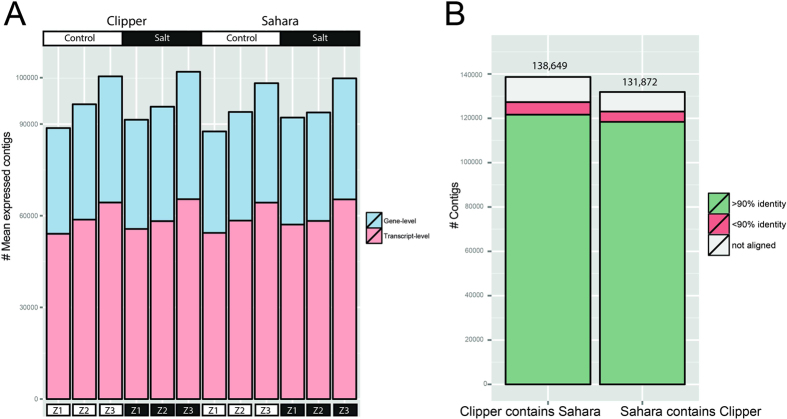
(**A**) Number of mean expressed contigs at the transcript- and gene-level; (**B**) Reciprocal cross-validation of the Clipper and Sahara assemblies. Blast-like alignment tool (BLAT^21^) was used for bi-directional sequence similarity analysis. Total number of contigs is noted above the bars. Z1, meristematic zone; Z2, elongation zone; Z3, maturation zone.

The mean expressed contig number was between 54,000 and 65,000 at the transcript level (between 33,000 and 41,000 at the gene level) in Clipper and Sahara, respectively, with the maturation zone (Z3) having a slightly higher number of expressed contigs in both genotypes compared to the other two zones (Fig. 1A).

To assess sequence similarity between genotypes, we performed a bi-directional sequence similarity analysis (see Methods) using Blast-like alignment tool (BLAT^21^). As shown in Fig. 1B, over 87% of Clipper contigs have similarity to Sahara with at least 90% identity. For Sahara contigs, over 89% of contigs had the highest scoring similarity to a Clipper contig with at least 90% identity.

### Comparative genomics with existing genome-based resources

**Figure 2:**
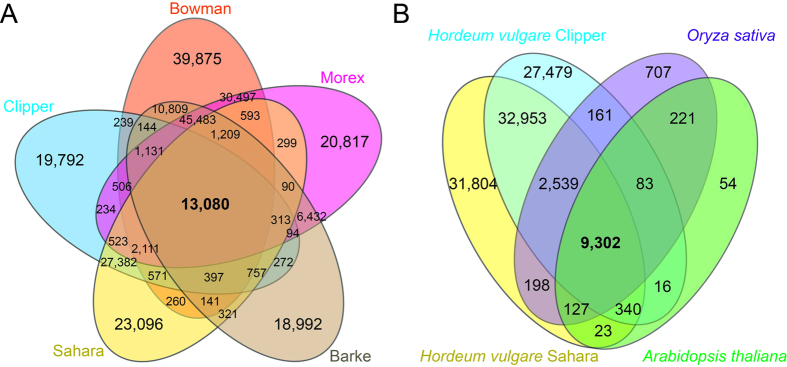
Transcript sequence comparisons of *Hordeum* genotypes and between other plant species. (**A**) Transcript sequence comparison of transcriptomes of five *Hordeum* genotypes. Distribution of orthologous groups (OG) amongst barley genotypes from both the MIPS consortium and *de novo* sequencing. (**B**) Transcript sequence comparison between *Hordeum* genotypes Clipper and Sahara, and a model eudicot (*Arabidopsis thaliana*), as well as a model monocot (*Oryza sativa*) transcriptomes. OrthoFinder (v0.2.8^23^) was used for identification of orthologous groups. Shape and colour used for each species is noted with the total number of shared OGs between each species.

To characterize and approximate the coverage of sequenced and assembled transcripts representing common gene loci to further support transcriptome completeness of the *de novo* assembly, we performed pairwise BLASTP of Clipper and Sahara predicted proteomes to thirteen angiosperms using NCBI BLAST+^22^. Transcriptomes of Clipper and Sahara show widespread orthologous groups to the Munich Information Center for Protein Sequences (MIPS) barley cultivar assemblies (Fig. 2A) using OrthoFinder^23^. A total of 317,262 orthologous groups (OGs) involved one or more of the five barley genotypes. Of these, Clipper contigs were part of 72,873 OGs and Sahara contigs participated in 77,286 OGs. A total of 80,039 Clipper contigs, about 58% of contigs in the transcriptome, were part of an OG, with a similar number found in Sahara (79,885 Sahara contigs were part of an OG, about 61%). 13,080 OGs contained predicted proteins from all five *Hordeum* genotypes.

In addition, we performed sequence comparisons to identify relationships between Clipper and Sahara to dicot (*Arabidopsis thaliana*) and monocot model plants (*Oryza sativa*) (Fig. 2B) as well as other plant species with key evolutionary relationships obtained from Phytozome^24^. This included the most ancient angiosperm *Amborella trichopoda,* the emerging model plant *Aquilegia coerulea,* the tree species models *Eucalyptus grandis* and *Populus trichocarpa*, the legume model *Medicago truncatula*, and as well the *Poaceae* species *Zea mays, Sorghum bicolor*, and *Brachypodium distachyon.* The highest number of hits was detected for the Clipper and Sahara genotypes against *Hordeum vulgare* cvs Barke, Bowman, and Morex genomes (Table 2). The quality of the annotation was supported by the observation that 70–95.8% of the *ab initio* predicted protein sequences of Clipper and Sahara had at least one ortholog in any of the three *Poaceae* genomes present in Phytozome (*Zea mays*, *Sorghum bicolor*, or *Brachypodium distachyon*) (Table 2).

### Validation of the presence of key salt-responsive genes

To further support the transcriptome completeness of the *de novo* assembly, we identified four putative *Oryza sativa High Affinity Potassium Transporter* (*OsHKT1.5*, *OsHKT2.1*, *OsHKT2.3*, and *OsHKT2.4*) orthologs sharing 62.35–72.09%, and four putative *Arabidopsis thaliana Salt Overly Sensitive* (*AtSOS1*, *AtSOS2*, *AtSOS3*, and *AtSOS4*) orthologs sharing 60.87–78.25% sequence identity in the cv. Clipper and LR Sahara assemblies that were differentially expressed along the root zones (Supplemental Figs S1 and S2).

In rice, the stelar Na^+^ transporter encoding gene *OsHKT1.5* was found to be expressed at higher levels after salt treatment in salt-tolerant rice lines^25^. Here, both *OsHKT1.5* and *OsHKT2.4* orthologs found in cv. Clipper and LR Sahara were only expressed after salinity stress (mature zones), and were not detectable under control conditions (Supplemental Fig. S1). *OsHKT2.3* detected in Clipper showed increased expression in the elongation zones both before and after salinity stress, and in the maturation zone exclusively after salinity stress, whereas *OsHKT2.1* showed higher transcript levels before salinity stress particularly in the elongation and maturation zones, consistent with both microarray and quantitative PCR results reported in ref. 25.

In *Arabidopsis,* the *SOS2* gene is required for intracellular Na^+^ and K^+^ homeostasis, and although expression is present in the root without treatment, *SOS2* expression is up-regulated by salt stress^26^. *AtSOS2* orthologs (with similar trends for *AtSOS4*) detected in both cv. Clipper and LR Sahara were found at increased levels in the elongation zone under both conditions, and at increased levels after salinity in the maturation zone (Supplemental Fig. S2). By contrast, *AtSOS1* and *AtSOS3* orthologs were expressed more evenly across the root zones. Sahara expressed all four detected *AtSOS* orthologs at higher levels compared to Clipper.

### Gene ontology analysis of barley transcripts

The assembled barley transcriptomes were annotated using BLASTP to UniProtKB/SwissProt, and BLASTX to UniProtKB/TrEMBL entries pre-loaded into Trinotate (https://trinotate.github.io/). For both genotypes, we were able to annotate two thirds of the assembled transcriptome: BLASTP yielded 76,269 (cv. Clipper) and 76,276 (LR Sahara) hits, and BLASTX resulted in 71,936 (cv. Clipper) and 72,451 (LR Sahara) hits, with 88,831 (cv. Clipper) and 88,017 (LR Sahara) hits combined. To investigate the biological role of genes regulated by salinity stress in both Clipper and Sahara, we tested for enriched GO (Gene Ontology) terms for both transcriptomes for differentially expressed (DE) contigs (log_2_-fold change > 1, FDR < 0.05), which provide a detailed annotation of gene function, biological process and cellular location of the gene product (Table 3; Supplemental Figs S3 and S4, cv. Clipper and LR Sahara, respectively).

GO analysis indicated that a total of 24,670 Clipper contigs were associated with biological process GO annotations, of which about one-third (8,919) were differentially expressed (DE) in one or more comparisons (see Methods; Supplemental Fig. S3). For Sahara, 24,825 contigs had biological process GO annotations, of which 9,268 contigs were considered DE in one or more comparison (Supplemental Fig. S4). The molecular function and cellular component GO annotations contained a similar number of GO terms as well as contigs associated with these processes which were significantly differentially expressed. GO analysis indicated that a total of 9,286 and 8,600 Clipper contigs were associated with molecular function or cellular component GO annotations and were DE in one or more comparisons (see Methods; Supplemental Fig. S3). For Sahara, 9,794 and 8,931 contigs had molecular function or cellular component GO annotations, respectively, and were considered DE in one or more comparisons (Supplemental Fig. S4).

**Figure 3:**
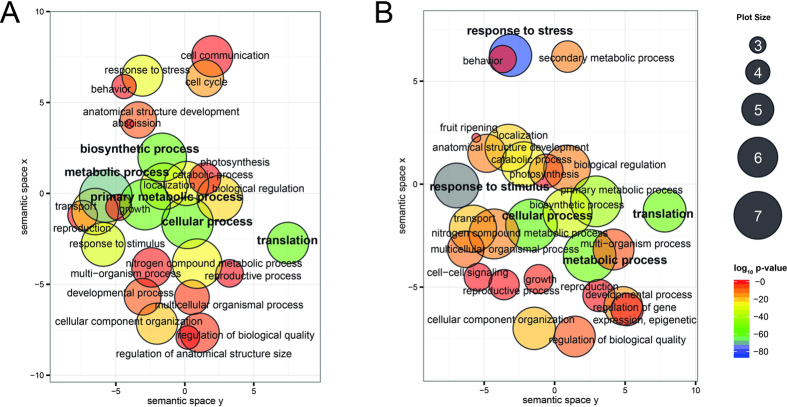
GO enrichment analyses summarized and visualized using REVIGO. (**A**) Significantly enriched GO terms related to biological processes in barley cv. Clipper after salinity treatment. (**B**) Significantly enriched GO terms related to biological processes in barley LR Sahara after salinity treatment. GO terms (Plant GO slims) are represented by circles and are clustered according to semantic similarities to other GO terms in the gene ontology (more general terms are represented by larger size circles, and adjoining circles are most closely related). Circle size is proportional to the frequency of the GO term, whereas color indicates the log_10_P-value for the enrichment derived from the AgriGO analysis (red higher, blue lower). The top five statistically most significant GO terms are highlighted in bold. Full data sets are available for cv. Clipper (Supplemental Table S3) and LR Sahara (Supplemental Table S4).

Enriched gene ontology term annotations were identified using AgriGO (http://bioinfo.cau.edu.cn/agriGO/) enrichment analysis supplemented by REVIGO (http://revigo.irb.hr/) visualization toolbox contigs after salinity stress for both Clipper (Fig. 3A) and Sahara (Fig. 3B). Differentially expressed contigs with biological process GO annotations were used as the query list as they were the best represented and the complete set of contigs with GO annotations (biological process, molecular function, and cellular component) was used as the background.

Statistically enriched terms gave insights into biological pathways that were likely to be highly active by comparing them to the frequency at which those GO terms appear in the transcriptome. Metabolic process, cellular process, biosynthetic process, primary metabolic process, and translation were the five most significantly enriched GO terms in Clipper after salinity stress; 54 GO terms were reported as statistically significant (p < 0.05) for enrichment in the Plant Slim GO analysis with AgriGO when comparing control versus salinity (Supplemental Table S3). In Sahara, a total of 55 biological processes were reported as statistically significant enriched biological processes, with response to stimulus, response to stress, cellular process, metabolic process, and translation the five most significant GO terms (Supplemental Table S4).

### Visualization of spatial gene expression profiles in barley roots

To highlight genes that have changed significantly in abundance along the different zones (Z1, Z2, Z3) of the root in both barley genotypes before and after salinity stress, we used a number of generalized linear models (GLM) to test for multiple interactions (treatment x zones) for both genotypes separately. Table 4 summarizes the GLM analysis, and Table 5 lists the top-ranked differentially expressed contigs after salinity stress per root zone with putative annotation. The full GLM data set is provided in Supplemental Data Sets S1 and S1, and DE gene annotation for cv. Clipper and LR Sahara, respectively, are provided in Supplemental Data Sets S3 and S4.

Surprisingly, only relatively few DE genes were detected as a response to treatment (100 mM NaCl) within individual root sections: In the meristematic zone (Z1), only 94 contigs (cv. Clipper) and 1,482 contigs (LR Sahara), respectively, were differentially expressed after salinity stress, with 8 and 24 times as many contigs up- than down-regulated in Clipper and Sahara, respectively. Examples include Dehydrin 5 (+5.3 log_2_ fold, cv. Clipper), a dehydration-inducible protein, and the sugar transporter SWEET (+8 log_2_ fold, LR Sahara). By contrast, in the elongation zone (Z2), 838 DE contigs (cv. Clipper) and 623 contigs (LR Sahara) were differentially expressed, with three times more genes up-than down-regulated in Sahara. Laccase, strongly up-regulated in the root tip in Clipper after salinity stress (+2.8 log_2_ fold), was strongly down-regulated in the elongation zone by −6 (log_2_)-fold. In addition, BBM2, a transcription factor that promotes cell proliferation, differentiation and morphogenesis^27^) (+5.3 log_2_ fold, cv. Clipper), and Pathogenesis-related Protein (+6.9 log_2_ fold, LR Sahara) were found to be strongly up-regulated after salinity stress in the elongation zone.

The largest treatment-specific transcriptional responses were observed in the maturation zone (Z3), with many more genes showing a significantly lower expression in this root zone after salinity treatment in both genotypes (cv. Clipper: DE contigs: 220 up and 1,340 down; LR Sahara: DE contigs: 676 up and 1,696 down). In both Clipper and Sahara, Cellulose synthase-like CslF9 transcripts were present at significantly lower levels in the maturation root zone after salinity stress (−6.5 and −5.7 log_2_ fold, respectively). Strikingly, contigs putatively annotated as peroxidases were expressed at much higher levels after salinity in the maturation root zone of LR Sahara (+5 log_2_ fold) compared to cv. Clipper (−6.9 log_2_ fold).

**Figure 4:**
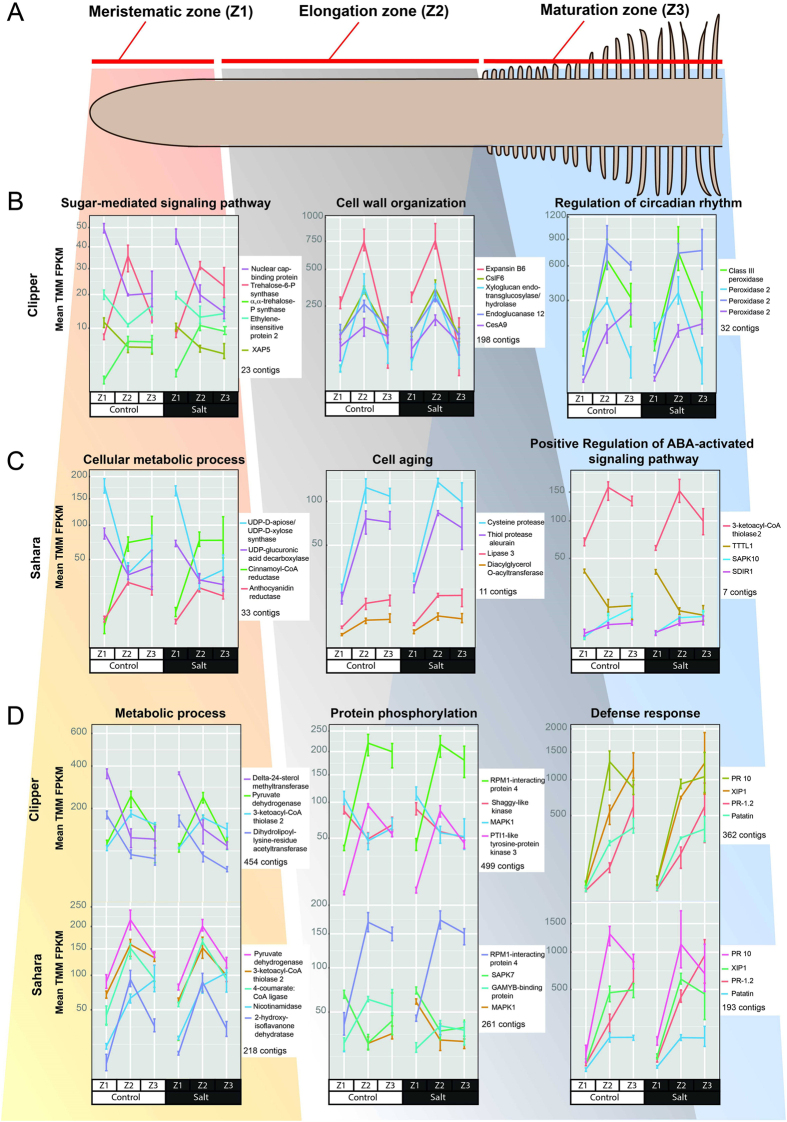
Transcriptomic analyses of barley cv. Clipper and LR Sahara across different zones of the root. (**A**) Model of a barley root, indicating longitudinal sections of three key developmental root zones. (**B**) Three major GO term clusters of gene expression across the different root zones detected in barley cv. Clipper only. (**C**) Three major GO term clusters of gene expression across the different root zones detected in barley LR Sahara only. (**D**) Three major GO term clusters of gene expression across the different root zones detected in both barley genotypes. Clusters consist of contigs found to be differentially expressed in any statistical comparison, and are reported by the number of contigs that are differentially expressed >2-fold and are annotated with the given GO term. Clusters are further grouped based on most contigs and highest mean TMM-normalised FPKM values present in the meristematic zone (Z1), elongation zone (Z2), or maturation zone (Z3), respectively (for D based on Clipper). Lines (colour depending on contig annotation, either the top four or five depicted based on highest expression value in the cluster) connect mean TMM-normalised FPKM values, with error bars representing the minimum and maximum TMM-normalised FPKM value. Text beneath gene ID list denotes the number of total contigs per cluster. Putative gene annotations were obtained using BLASTX on the UniRef90 database (www.uniprot.org) for the contigs for both transcriptomes. For the complete dataset see Supplemental Data Sets1–4. TMM, trimmed mean of M-values; FPKM, fragments per kilobase of transcript per million mapped reads.

The largest transcriptional differences in terms of magnitude and quantity were observed between the different root zones: More than 18,000 contigs were differentially expressed in both genotypes in the elongation zone (Z2) and maturation zone (Z3) compared to the meristematic zone (Z1), with similar numbers for both control and salinity conditions (Table 4). To investigate these spatial transcriptional differences in more detail, we identified differentially expressed contigs for each genotype with GO annotation (biological process), and putatively annotated them using Trinotate (Fig. 4). We detected a total of 712 unique GO terms for transcripts from cv. Clipper, 157 GO terms for LR Sahara, and detected a further 1,303 GO terms shared by DE transcripts from both genotypes. GO term clusters consist of contigs found to be differentially expressed in any statistical comparison (treatments, genotypes, and root zones; for details see Methods), and are reported by the number of contigs that are differentially expressed >2-fold and are annotated with the given GO term. As the total number of computed clusters exceeded 3,400 for both genotypes, only the most highly expressed contigs of clusters that contained the largest number of contigs are presented in Fig. 4. Clusters are further grouped based on number of contigs and highest mean Trimmed mean of M-values (TMM)-normalized FPKM values present in each of the zones: meristematic zone (Z1), elongation zone (Z2), or maturation zone (Z3), respectively (Fig. 4A, see also Methods). GO term-enriched clusters that were exclusively found in cv. Clipper (Fig. 4B), LR Sahara (Fig. 4C), and in both genotypes (4D) are visualized separately.

As expected, expression profiles differ between root zones reflecting their biological function, and also show differences between the two barley genotypes. In cv. Clipper, DE transcripts of the meristematic zone (Z1) are enriched in GO terms related to the sugar signaling pathway, whereas DE transcripts of both the elongation (Z2) and maturation zones (Z3) in cv. Clipper are enriched in GO terms related to cell wall organization and regulation of circadian rhythm, respectively (Fig. 4B). GO terms related to cell wall organization contained 198 transcripts that were putatively identified as cellulose synthase-like (*CslF*) and cellulose synthase catalytic subunit (*CesA9*) genes, which are part of the cellulose synthase superfamily involved in the biosynthesis of mixed linkage β-(1,3; 1,4)-glucans and cellulose (a β-(1,4)-glucan), respectively^28^, and highly expressed in the elongation zone of cv. Clipper both before and after salinity stress. In both the maturation and elongation zone, several peroxidases related to regulation of circadian rhythm show spatial differential expression, with several also showing expression increases in response to salt treatment.

In LR Sahara, the cellular metabolic process GO term cluster includes 33 transcripts with many of them showing the highest expression in the root tip, including transcripts encoding proteins involved in cell wall polysaccharide metabolism (UDP-D-apiose/UDP-D-xylose synthase, UDP-glucuronic acid decarboxylase) (Fig. 4C). By contrast, lignin (Cinnamoyl-CoA reductase) and flavonoid (anthocyanidin reductase) biosynthesis transcript abundance is very low in the root tip, and increase strongly towards the elongation and maturation zone. Transcripts related to cell aging were found to encode lipase 3, several proteases showing different spatial expression patterns, as well as the serine/threonine protein kinase SAPK10, and *Arabidopsis thaliana* Salt- and Drought-Induced Ring Finger 1 (SDIR1) were present at high levels in both the elongation and maturation zones. By contrast, transcripts involved in osmotic stress-responsive abscisic acid signaling such as Tetratricopeptide Repeat-Containing Protein (TTL1) were highly expressed only in the elongation zone^29^,^30^,^31^.

Most GO term assignments (1,303) contained transcripts from both cv. Clipper and LR Sahara (Fig. 4D). Transcripts assigned to the GO term metabolic process were particularly highly expressed in the root tip of cv. Clipper and the elongation zone of Sahara, including pyruvate dehydrogenase and 3-ketoacyl-CoA thiolase 2. Transcripts related to protein phosphorylation were present in both cv. Clipper and LR Sahara, and proteins involved in stress resistance such as RPM1-interacting protein 4 (RIN4) and mitogen-activated protein kinase (MAPK1) showed similar expression patterns in the root of both genotypes. In the maturation zone, defense-related transcripts were widespread, including: pathogenesis-related protein 10 (PR10), patatin, and Xylanase inhibiting protein XIP1 shown to be specifically induced in roots by biotic and abiotic stresses^32^,^33^,^34^.

## Discussion

In this study we generated novel root transcriptomic resources obtained from RNA of three different root zones, and created two *de novo* assemblies of a barley landrace and a malting cultivar with known contrasting responses to salinity. We have detected root-zone specific transcriptional signatures and dominant expression patterns along longitudinal axes of roots under salinity that often coincided with defined developmental zones and were distinct between cv. Clipper and LR Sahara.

Salinity has a negative impact on root growth in many plant species, including barley which is the most salt-tolerant cereal crop^35^ and thus a good model to study salt-tolerance mechanisms. The early phase of salinity stress is described as osmotic stress which leads to loss of turgor due to cell dehydration, as well as reduced rates of root cell division and expansion^36^. The barley malting cv. Clipper and the North African LR Sahara have contrasting root growth phenotypes in response to the early phase of salinity stress: In a previous study, the length of the cell division region in the meristematic zone was unaffected by salt treatment, and the cortical cells in the elongation zone continued to expand in the more salt-tolerant Clipper^37^. In addition, Clipper was shown to accumulate amino acids, sugars, and organic acids in a root-zone-specific manner hypothesized to be contributing to elongation in response to salt stress: In the meristematic zone, high levels of amino acids, but low levels of metabolites of the energy metabolism (TCA cycle and sugars) where detected, whereas both the elongation and maturation zones accumulated amino acids, sugars, and organic acids after salt treatment.

In our study we detected transcriptomics responses that reflect these physiological and metabolic changes. Similar to the previously observed metabolic response, the transcriptional expression gradient along the root in this study also shows more distinct responses for the meristematic zone than for the other two zones (Table 4). The differential gene expression analysis demonstrates that in cv. Clipper, several highly expressed genes at the root tip were associated with sugar-mediated signaling and osmotic stress response (Fig. 4). After salinity stress, Dehydrin 5, a protein involved in frost tolerance in barley^38^ and also enhancing tolerance to salt and osmotic stress in *Arabidopsis thaliana*^39^, showed an up-regulation of +5.3 (equivalent to +40 fold) (Table 5). Its high transcript abundance in the more salinity-tolerant genotype Clipper points towards a protective role against salinity stress. The elongation zone has the highest expression of genes involved in cell wall organization and protein phosphorylation: The AP2-like ethylene-responsive transcription factor BABY BOOM (BBM) 2 and agglutinin isolectin 2 were strongly up-regulated in the elongation zone after salinity stress in cv. Clipper, but both were strongly down-regulated at similar magnitude in the same zone in LR Sahara (Table 5). Members of the AP2 family of transcription factors such as BBM play important roles in cell proliferation and embryogenesis in *Arabidopsis thaliana*^40^, showing that pathways were activated in response to salinity to maintain cell expansion, a result in line with the higher root elongation rate in Clipper compared to Sahara^15^. Plant lectins are carbohydrate-binding glycoproteins that bind with free sugar or with sugar residues, which would be present at high levels in the elongation zone in Clipper after salinity stress^37^, and are known to play important roles in plant defense^41^ and are hypothesized to be induced by abiotic stresses^42^. Genotype-specific transcriptional changes were also present in both the maturation and elongation zones, such as several peroxidase transcripts which were found at increased levels after salinity stress in cv. Clipper, suggesting that Clipper is capable of up-regulating its root antioxidant system in response to salinity stress (Fig. 4).

In stark contrast to cv. Clipper, which is able to maintain root elongation, cell division and overall root growth, Sahara’s root growth is severely limited under short-term salt stress^15^. Under short-term stress conditions, cell division is inhibited in the root tip, whereas cell expansion increases in the elongation zone, but overall, root growth is more inhibited in Sahara compared to Clipper^37^. In addition, Sahara showed lower levels of amino acids, sugars, and organic acids in both the root tip and the elongation zone, and higher levels in the maturation zone similar to Clipper in response to salinity stress^37^. This was reflected in the transcriptomic response as many more transcriptional changes after salinity stress were detected in the meristematic zone of Sahara compared to Clipper, indicating a much stronger impact of salt on Sahara roots compared to Clipper (Table 4). Gene expression analysis showed that highly expressed genes at the root tip are associated with cellular metabolic processes in LR Sahara. As an example, the sugar transporter SWEET showed an upregulation of +8-log_2_-fold (equivalent to +256-fold) indicating the activation of sugar signaling pathways that interact with stress pathways to modulate carbohydrate metabolism, possibly a response to low sugar levels in the root tip after salinity stress (Table 5). The transcriptomic profile confirms that Sahara is more affected by salt in the initial stages of root growth, resulting in up-regulation of cell senescence-related transcripts, such as proteases and lipases in the elongation zone. The adaptive process to abiotic stress is primarily regulated by ABA, and the greater impact of salinity on the sensitive LR Sahara compared to cv. Clipper was also reflected by the high level of differentially expressed transcripts related to ABA signaling in the maturation zone of Sahara (Fig. 4). Transcripts involved in plant defense such as patatin, a storage glycoprotein with lipase activity found in *Solanum tuberosum*^43^, were common between both genotypes and expressed in the elongation and maturation zones after salinity stress.

Transcriptomic analyses provide powerful tools to investigate the molecular basis of plant responses to stress and previous analyses in *Arabidopsis thaliana* and rice highlighted significant differences in transcript abundance and identity between different cell types of roots using microarray analyses^6^,^44^. Our findings emphasize that the different barley root zones are highly specialized in their biological function and although this is based on RNA expression, recent studies on spatially resolved root metabolism^45^,^46^ and proteins^47^ support this notion. Using this comprehensive root transcriptome dataset, we have demonstrated that transcriptional differences in different root zones within a species are much larger than between treatments. When either whole organs or entire plants are analyzed, the results provide only averaged responses that may be highly variable across different regions of the plant. This is of particular concern when investigating salinity stress at the seedling stage because of the developmental gradient that occurs within growing regions of the roots. Further studies will determine if spatially defined alternative splicing events, such as differential intron retention, are induced by salinity stress as part of a root-zone-specific adaptation response to salinity in barley.

Taken together, our study provides detailed insights into the barley root transcriptome that has allowed us to identify and annotate transcripts associated with specialized development and salinity stress response functions in different developmental root zones in two distinct barley varieties. The availability of additional transcriptome reference sets from a barley landrace and a malting cultivar as presented here will serve as valuable resources for plant breeders and to the research community for further functional and comparative genomics studies.

## Methods

### Barley genotypes

Genotypes of barley (*Hordeum vulgare* L.) were originally sourced from the Australian Centre for Plant Functional Genomics (University of Adelaide). Two genotypes of barley, the malting variety Clipper (Australia) and the landrace Sahara 3771 (North Africa), were used for RNA-Seq analysis, and were selected based on previously reported physiological diversity in salt tolerance^13^,^15^. These genotypes are parents of a mapping populations (Clipper x Sahara 3771^48^), and genetic resources are available for further genetic characterisation.

### Sample preparation, RNA isolation, and Illumina sequencing

Uniformly sized seeds were selected, surface-sterilised, and grown under control (nutrient medium without additional NaCl) and salt-treated (nutrient medium supplemented with 100 mM NaCl) conditions as described in ref. 15. After three days of germination, seminal roots were dissected, collected into 1.5 mL tubes, immediately snap-frozen in liquid nitrogen, and then stored at −80 °C. Samples were dissected in the following steps: A 1.5 mm long section marked ‘Zone 1’ (meristematic zone) was taken from the root tip. The second section (‘Zone 2’) was dissected from the elongation zone up to the third section, ‘Zone 3’ (maturation zone), which was excised at the point of visible root hair elongation up to ¾ of the entire root. Four biological replicates were generated for each sample in four separate experiments totaling 48 samples. Total RNA was isolated from 50 mg root tissue using the Qiagen RNeasy kit following the manufacturer’s protocol. The RNA was analyzed for quality and concentration using a DeNovix DS-11 spectrophotometer (Wilmington, DE, USA) and an Agilent Technologies 2100 Bioanalyzer. Libraries were amplified through 13 cycles of PCR using Illumina guidelines. All RNA-seq libraries were constructed and paired-end sequenced (100 bp) on an Illumina HiSeq 2000 system at the Australian Genome Research Facility (Melbourne, Australia). Four lanes were used for each genotype, and all 48 samples were run on a single flow cell. The Illumina TruSeq RNA Sample preparation kit v2 (Illumina Inc.) was used according to the manufacturer’s protocol. Image analysis was performed in real time by the HiSeq Control Software (HCS) v1.4.8 and Real Time Analysis (RTA) v1.12.4.2, running on the instrument computer. The RNA was sequenced to a depth of approximately 31 million read-pairs per sample per lane, giving a total of 1.48 billion reads (749 million read-pairs), yielding 296.06 Gb data. The Illumina CASAVA (Consensus Assessment of Sequence And Variation) 1.8 pipeline was used to generate the sequence data.

### Bioinformatics analysis

#### Pre-processing of reads

All read pairs were trimmed for quality using Nesoni software (https://github.com/Victorian-Bioinformatics-Consortium/nesoni; q = 20, Illumina TruSeq Adaptor primers removed, min length 30 bp, singletons discarded), and technical replicates of each condition in four lanes were pooled to form a single dataset per experimental condition. FastQC software (http://www.bioinformatics.bbsrc.ac.uk/projects/fastqc/) and manual inspection of each sample were used to assess the quality of raw sequence data and results of quality trimming.

#### De novo transcriptome assembly

Data redundancy and error reduction was performed using digital normalization (Diginorm), which identified approximately 80% of the reads^49^ as redundant and which were subsequently excluded from the assembly. All biological replicates (per genotype) were pooled into one paired-end read set for independent *de novo* assemblies per genotype. *De novo* transcriptome assembly was performed at the Victorian Life Sciences Computation Initiative (VLSCI, University of Melbourne) using 64 GB memory and 16 cores, using the Trinity (v20140717) assembler available at http://trinityrnaseq.github.io/ 16, with k = 25.

To assess the completeness of each *de novo* assembled transcriptome, the Core Eukaryotic Genes Mapping Approach (CEGMA) program v2.4.010312^18^ was used. The most likely longest ORF (open reading frame) peptide candidates were extracted from each transcriptome using TransDecoder with default parameters (http://transdecoder.github.io).

Annotation of each transcriptome was performed using the Trinotate pipeline (v2.0.1, http://trinotate.github.io/) including all programs except RNAmmer. BLASTP results to the SwissProt database, and BLASTX results to the UniRef90 database (www.uniprot.org 50) were obtained for all contigs for both transcriptomes. To obtain GO (Gene Ontology) annotations for both transcriptomes, we used the Trinotate-integrated UniProtKB GO annotations.

#### Quantitation of gene expression

Each sample was individually aligned using Bowtie v2.1.0^51^ to its respective transcriptome with TPM (transcripts per million), FPKM (Fragments Per Kilobase Of Exon Per Million Fragments Mapped), credibility intervals and expected counts produced using RSEM v1.2.19^52^ via the align_and_estimate.pl perl script, which is part of the Trinity software suite. Both *de novo* trinity component (“gene”) and transcript contig expression matrices were computed. Cross-sample normalization of FPKM values was carried out using Trimmed mean of M-values (TMM) normalization available in the edgeR software package (version v3.8.6^53^,^54^.

#### Identification of outlier samples

Dimensionality reduction to two dimensions using MDS (multidimensional scaling) of the TMM-normalised FPKM matrix was then performed to observe the similarity between samples within each genotype (data not shown). This revealed that one biological replicate sample of Clipper control maturation zone was significantly different to other replicates of the same condition, therefore was excluded from all subsequent analyses.

#### Identification of differentially expressed genes

Transcript isoform-level expected counts matrices for each genotype were obtained from RSEM and passed to R v3.1.1^55^ and edgeR (version v3.8.6^53^,^54^) to be used for differential expression analysis. Only matrix rows containing four or more replicates with counts per million (CPM) greater than three were retained for the analysis. We conducted generalized linear model (GLM) analyses for each genotype, and used a number of models for multiple comparisons between treatments, genotypes, and root zones: (i) a basic model treating treatment and zone as separate variables; (ii) an interaction model considering the interaction of zone within treatment, and (iii) an interaction model considering the interaction of treatment within zone. Common-, trended-, and tagwise-dispersion estimates were calculated for each model, and a Benjamini-Hochberg false discovery rate (FDR) of 0.05 or less and an absolute log_2_-fold change greater than 1 was required to define a gene as differentially expressed. We visualized the TMM-normalised FPKM values of differentially expressed contigs for each cultivar with GO annotation using ggplot2^56^ as provided in Fig. 4 of the Results section and Supplemental Data Sets 3 and 4 to identify contigs from GO terms that warrant further investigation.

#### Gene Ontology and Pathway enrichment analysis

Singular enrichment analysis (SEA) on the GLM results was performed using AgriGO (http://bioinfo.cau.edu.cn/agriGO/analysis.php) for each genotype separately, using results from the GLM differential expression analysis. For each separate genotype analysis, we employed the following AgriGO SEA parameter settings: hypergeometric test, with Yekutieli multi-testing adjustment, 0.05 significance level, 5 minimum mapping entries and Plant Slim GO and biological process gene ontology. REVIGO^57^; http://revigo.irb.hr/, default settings except small results requested) was applied to visualize the summary results and generate Fig. 3.

#### Identification of orthologous genes

We used OrthoFinder^23^; available from http://www.stevekellylab.com/software/orthofinder, v0.2.8) using default parameters to identify orthologous gene groups from 15 angiosperms; these included five *Hordeum* genotypes (Clipper, Sahara and independently sequenced Barke, Morex and Bowman) along with key angiosperms (such as *Amborella trichopoda*, *Arabidodopsis thaliana* and *Oryza sativa*) obtained from Phytozome (www.phytozome.net version 10.2, accessed May 2015; 25) to enable comparison of the gene complement at key points in evolution. Augustus v2.5.5^58^ was used to construct *ab initio* predicted proteomes unless an annotated genome prediction was already available, with a number of plant species gene models including *Arabidopsis thaliana*, *Oryza sativa*, and *Zea mays* used as references. Pairwise species blast was performed using the BLAST v2.2.29+ downloaded from NCBI (National Centre for Biotechnology Information, http://www.ncbi.nlm.nih.gov/).

## Additional Information

**How to cite this article**: Hill, C. B. *et al*. *De novo* transcriptome assembly and analysis of differentially expressed genes of two barley genotypes reveal root-zone-specific responses to salt exposure. *Sci. Rep.* **6**, 31558; doi: 10.1038/srep31558 (2016).

## Supplementary Material

Supplementary Information

Supplementary Tables

Supplementary Data Set 1

Supplementary Data Set 2

Supplementary Data Set 3

Supplementary Data Set 4

## Figures and Tables

**Table 1:** A comparison of *Hordeum vulgare* species Clipper and Sahara transcriptome statistics.

	Cv. Clipper	LR Sahara
**# Assembled contigs**	138,649	131,872
**# Trinity components/with multiple isoforms**	100,228/15,919	92,123/15,443
**N50**	24,876	24,473
**L50**	1,752	1,886
**Trimmed reads (q = 20)**	585,745,455	605,194,147
**Longest contig (bp)**	16,480	16,481
**Shortest contig (bp)**	201	224

Mean number of features with FPKM > 1 (n = 4). Bp, base pairs; Cv., cultivar; LR, landrace; FPKM, fragments per kilobase of transcript per million mapped reads; q, quality threshold; Z1, meristematic zone; Z2, elongation zone; Z3, maturation zone.

**Table 2:** A summary of the comparison of *ab initio* predicted proteomes between *Hordeum* and other plant species.

Plant species*	Cv. Clipper		LR Sahara		Reference
	#hits	%hits	#hits	%hits	
*Amborella trichopoda*	18,574	69.2	18,604	69.3	59
*Aquilegia coerulea*	21,480	86.5	21,534	86.8	*Aquilegia coerulea* Genome Sequencing Project, http://phytozome.jgi.doe.gov/
*Arabidopsis thaliana*	23,111	84.3	23,166	84.5	60
*Brachypodium distachyon*	25,106	94.6	25,151	94.7	61
*Eucalyptus grandis*	30,554	84.0	30,582	84.1	62
*Hordeum vulgare cv. Barke*	103,719	61.7	101,799	60.5	63
*Hordeum vulgare cv. Bowman*	119,845	52.6	118,472	52.0	63
*Hordeum vulgare cv. Morex*	109,745	57.2	108,741	56.7	63
*Medicago truncatula*	25,115	56.9	25,154	57.0	64
*Oryza sativa*	28,446	72.8	28,534	73.1	65
*Populus trichocarpa*	34,478	83.4	34,489	83.4	66
*Sorghum bicolor*	26,419	95.7	26,442	95.8	67
*Zea mays*	62,166	70.0	62,311	70.2	68

OrthoFinder (v0.2.8^23^) species pair-wise BLASTP protein sequence comparisons of *Hordeum vulgare* cv. Clipper and LR Sahara were used against other sequenced plant species. Source: Phytozome (http://www.phytozome.net version 10.2). Augustus (v2.5.5) was used to construct *de novo* predicted proteomes unless proteome predictions were already available. Pairwise species blast was performed using NCBI BLAST+ v2.2.29.

**Table 3:** Summary statistics of the gene ontology analysis of barley cv. Clipper and LR Sahara transcripts.

	Cv. Clipper	LR Sahara
# Assembly contigs	138,649	131,872
GLM-reported DE contigs (any comparison)	20,015	20,344
DE contigs with GO annotation/unique GO terms	10,857/4,654	11,211/4,704
DE contigs with biological process GO annotation/unique GO terms	8,919/2,578	9,268/2,569
DE contigs with molecular function GO annotation/unique GO terms	9,286/1,547	9,794/1,589
DE contigs with cellular component GO annotation/unique GO terms	8,600/529	8,931/546

DE, differentially expressed; GLM, generalized linear model; GO, gene ontology.

**Table 4:** Summary statistics of number of differentially expressed contigs as determined by GLM analysis.

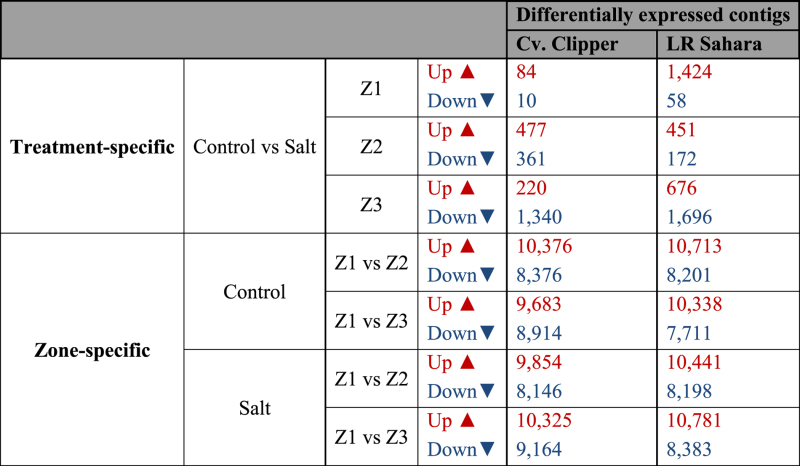

Transcript isoform-level expected counts matrices for each genotype were obtained from RSEM and passed to R v3.1.1^52^ with the edgeR (version v3.8.6) package used for differential expression analysis. Only matrix rows containing four or more samples with counts per million (CPM) greater than three were retained for the analysis. A Benjamini-Hochberg false discovery rate (FDR) of 0.05 or less and an absolute log_2_-fold change greater than 1 was required to define a gene as differentially expressed (DE). GLM, generalized linear model; Z1, meristematic zone; Z2, elongation zone; Z3, maturation zone. The full data sets are provided in Supplemental Data Sets S1 (cv. Clipper) and S2 (LR Sahara).

**Table 5:** Annotated differentially expressed contigs after salinity stress in different root tissues as identified by RNA-seq.

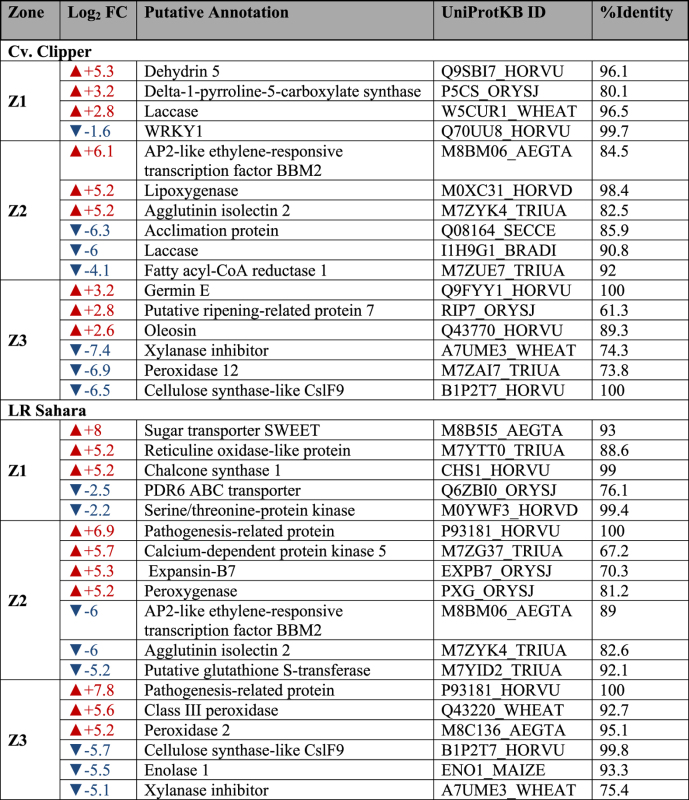

FC, fold change; Z1, meristematic zone; Z2, elongation zone; Z3, maturation zone. Full data sets are provided for cv. Clipper in Supplemental Data Sets S1 (full GLM model data set) and S3 (full annotation), and for LR Sahara in Supplemental Data Sets S2 (full GLM model data set) and S4 (full annotation).

## References

[b1] Glenn E. P., Brown J. J. & Blumwald E. Salt tolerance and crop potential of halophytes. Crit. Rev. Plant Sci. 18, 227–255 (1999).

[b2] Ouyang B. . Identification of early salt stress response genes in tomato root by suppression subtractive hybridization and microarray analysis. J. Exp. Bot. 58, 507–520 (2007). 17210988 10.1093/jxb/erl258

[b3] Ishikawa H. & Evans M. L. Specialized zones of development in roots. Plant Physiol. 109, 725–727 (1995). 11539165 10.1104/pp.109.3.725 PMC161370

[b4] Hochholdinger F., Woll K., Sauer M. & Dembinsky D. Genetic dissection of root formation in maize (*Zea mays*) reveals root‐type specific developmental programmes. Ann. Bot. 93, 359–368 (2004). 14980975 10.1093/aob/mch056 PMC4242335

[b5] Birnbaum K. . A gene expression map of the Arabidopsis root. Science 302, 1956–1960 (2003). 14671301 10.1126/science.1090022

[b6] Brady S. M. . A high-resolution root spatiotemporal map reveals dominant expression patterns. Science 318, 801–806 (2007). 17975066 10.1126/science.1146265

[b7] Moussaieff A. . High-resolution metabolic mapping of cell types in plant roots. Proc. Natl. Acad. Sci. USA 110, E1232–E1241 (2013). 23476065 10.1073/pnas.1302019110 PMC3612672

[b8] Dinneny J. R. . Cell identity mediates the response of Arabidopsis roots to salinity and iron stress. Science 320, 942–945 (2008). 18436742 10.1126/science.1153795

[b9] Van Hintum T. & Menting F. Diversity in *ex situ* genebank collections of barley in Diversity in Barley (Hordeum vulgare) (eds von Bothmer R. . ) 247–257 (Elsevier Science B. V., 2003).

[b10] Venturini L. . De novo transcriptome characterization of *Vitis vinifera* cv. Corvina unveils varietal diversity. BMC Genomics 14, 4 (2013). 23331995 10.1186/1471-2164-14-41 PMC3556335

[b11] Natera S. H., Hill C. B., Rupasinghe T. W. & Roessner U. Salt-stress induced alterations in the root lipidome of two barley genotypes with contrasting responses to salinity. Funct. Plant Biol. 43, 207–219 (2016). 10.1071/FP15253 32480454

[b12] Tavakkoli E., Rengasamy P. & McDonald G. K. The response of barley to salinity stress differs between hydroponic and soil systems. Funct. Plant Biol. 37, 621–633 (2010).

[b13] Widodo . Metabolic responses to salt stress of barley (*Hordeum vulgare* L.) cultivars, Sahara and Clipper, which differ in salinity tolerance. J. Exp. Bot. 60, 4089–4103 (2009). 19666960 10.1093/jxb/erp243 PMC2755029

[b14] Rivandi J. . A *SOS3* homologue maps to *HvNax4*, a barley locus controlling an environmentally sensitive Na^+^ exclusion trait. J. Exp. Bot. 62, 1201–1216 (2011). 21047983 10.1093/jxb/erq346 PMC3022402

[b15] Shelden M. C., Roessner U., Sharp R. E., Tester M. & Bacic A. Genetic variation in the root growth response of barley genotypes to salinity stress. Funct. Plant Biol. 40, 516–530 (2013). 10.1071/FP12290 32481128

[b16] Grabherr M. G. . Trinity: reconstructing a full-length transcriptome without a genome from RNA-Seq data. Nat. Biotechnol. 29, 644–652, 10.1038/nbt.1883 (2011). 21572440 PMC3571712

[b17] Haas B. J. . *De novo* transcript sequence reconstruction from RNA-Seq: reference generation and analysis with Trinity. Nat. Protoc. 8, 1494–1512, 10.1038/nprot.2013.084 (2013). 23845962 PMC3875132

[b18] Parra G., Bradnam K. & Korf I. CEGMA: a pipeline to accurately annotate core genes in eukaryotic genomes. Bioinformatics 23, 1061–1067 (2007). 17332020 10.1093/bioinformatics/btm071

[b19] Nakasugi K. . De novo transcriptome sequence assembly and analysis of RNA silencing genes of *Nicotiana benthamiana*. PloS One 8, e59534 (2013). 23555698 10.1371/journal.pone.0059534 PMC3610648

[b20] Zhang S. . De novo characterization of fall dormant and nondormant alfalfa (*Medicago sativa* L.) leaf transcriptome and identification of candidate genes related to fall dormancy. PloS One 10, e0122170–e0122170 (2014). 25799491 10.1371/journal.pone.0122170 PMC4370819

[b21] Kent W. J. BLAT–the BLAST-like alignment tool. Genome Res. 12, 656–664 (2002). 11932250 10.1101/gr.229202 PMC187518

[b22] Mount D. W. Using the basic local alignment search tool (BLAST). Cold Spring Harb. Protoc. 2007 (7), pdb-top17 (2007). 10.1101/pdb.top17 21357135

[b23] Emms D. M. & Kelly S. OrthoFinder: solving fundamental biases in whole genome comparisons dramatically improves orthogroup inference accuracy. Genome Biol . 16, 157, 10.1186/s13059-015-0721-2 (2015). 26243257 PMC4531804

[b24] Goodstein D. M. . Phytozome: a comparative platform for green plant genomics. Nucleic Acids Res . 40 (D1), D1178–D1186 (2012). 22110026 10.1093/nar/gkr944 PMC3245001

[b25] Cotsaftis O. . Root-specific transcript profiling of contrasting rice genotypes in response to salinity stress. Mol. Plant 4, 25–41 (2011). 20924028 10.1093/mp/ssq056

[b26] Liu J., Ishitani M., Halfter U., Kim C. S. & Zhu J. K. The *Arabidopsis thaliana SOS2* gene encodes a protein kinase that is required for salt tolerance. Proc. Natl. Acad. Sci. USA. 97, 3730–3734 (2000). 10725382 10.1073/pnas.060034197 PMC16308

[b27] Boutilier K. . Ectopic expression of BABY BOOM triggers a conversion from vegetative to embryonic growth. Plant Cell 14, 1737–1749 (2002). 12172019 10.1105/tpc.001941 PMC151462

[b28] Tanaka K. . Three distinct rice cellulose synthase catalytic subunit genes required for cellulose synthesis in the secondary wall. Plant Physiol . 133, 73–83 (2003). 12970476 10.1104/pp.103.022442 PMC196581

[b29] Kobayashi Y. . Abscisic acid‐activated SNRK2 protein kinases function in the gene‐regulation pathway of ABA signal transduction by phosphorylating ABA response element‐binding factors. Plant J. 44, 939–949 (2005). 16359387 10.1111/j.1365-313X.2005.02583.x

[b30] Rosado A. . The Arabidopsis tetratricopeptide repeat-containing protein TTL1 is required for osmotic stress responses and abscisic acid sensitivity. Plant Physiol . 142, 1113–1126 (2006). 16998088 10.1104/pp.106.085191 PMC1630727

[b31] Zhang Y. . SDIR1 is a RING finger E3 ligase that positively regulates stress-responsive abscisic acid signaling in Arabidopsis. Plant Cell 19, 1912–1929 (2007). 17573536 10.1105/tpc.106.048488 PMC1955734

[b32] Goesaert H., Gebruers K., Brijs K., Courtin C. M. & Delcour J. A. XIP-type endoxylanase inhibitors in different cereals. J. Cereal Sci. 38, 317–324 (2003). 10.1021/jf0262155 12797742

[b33] Jammes F. . Genome‐wide expression profiling of the host response to root‐knot nematode infection in Arabidopsis. Plant J. 44, 447–458 (2005). 16236154 10.1111/j.1365-313X.2005.02532.x

[b34] Hashimoto M. . A novel rice PR10 protein, RSOsPR10, specifically induced in roots by biotic and abiotic stresses, possibly via the jasmonic acid signaling pathway. Plant Cell Physiol . 45, 550–559 (2004). 15169937 10.1093/pcp/pch063

[b35] Maas E. V. & Hoffman G. J. Crop salt tolerance–current assessment. J. Irrig. Drain. E-ASCE 103, 115–134 (1977).

[b36] Munns R. & Tester M. Mechanisms of salinity tolerance. Annu. Rev. Plant Biol. 59, 651–681 (2008). 18444910 10.1146/annurev.arplant.59.032607.092911

[b37] Shelden M. C., Dias D. A., Jayasinghe N. S., Bacic A. & Roessner U. Root spatial metabolite profiling of two genotypes of barley (*Hordeum vulgare* L.) reveals differences in response to short-term salt stress. J. Exp. Bot. 37, 3731–3745 (2016). 26946124 10.1093/jxb/erw059 PMC4896359

[b38] Kosová K. . Expression of *Dehydrin 5* during the development of frost tolerance in barley (*Hordeum vulgare*). J. Plant Physiol. 165, 1142–1151 (2008). 18242771 10.1016/j.jplph.2007.10.009

[b39] Brini F. . Overexpression of wheat dehydrin *DHN-5* enhances tolerance to salt and osmotic stress in *Arabidopsis thaliana*. Plant Cell Rep . 26, 2017–2026 (2007). 17641860 10.1007/s00299-007-0412-x

[b40] El Ouakfaoui S. . Control of somatic embryogenesis and embryo development by AP2 transcription factors. Plant Mol. Biol. 74, 313–326 (2010). 20798978 10.1007/s11103-010-9674-8 PMC2952763

[b41] Chrispeels M. J. & Raikhel N. V. Lectins, lectin genes, and their role in plant defense. Plant Cell 3, 1–9 (1991). 1824332 10.1105/tpc.3.1.1 PMC159974

[b42] Zhang W. . Isolation and characterization of a jacalin-related mannose-binding lectin from salt-stressed rice (*Oryza sativa*) plants. Planta 210, 970–978 (2000). 10872230 10.1007/s004250050705

[b43] Mignery G. A., Pikaard C. S. & Park W. D. Molecular characterization of the patatin multigene family of potato. Gene 62, 27–44 (1988). 3371664 10.1016/0378-1119(88)90577-x

[b44] Takehisa H. . Genome‐wide transcriptome dissection of the rice root system: implications for developmental and physiological functions. Plant J. 69, 126–140 (2012). 21895812 10.1111/j.1365-313X.2011.04777.x

[b45] Walter A., Feil R. & Schurr U. Expansion dynamics, metabolite composition and substance transfer of the primary root growth zone of *Zea mays* L. grown in different external nutrient availabilities. Plant Cell Environ . 26, 1451–1466 (2003).

[b46] Peukert M. . Spatially resolved analysis of small molecules by matrix‐assisted laser desorption/ionization mass spectrometric imaging (MALDI‐MSI). New Phytol. 193, 806–815 (2012). 22126099 10.1111/j.1469-8137.2011.03970.x

[b47] Marcon C. . A high resolution tissue-specific proteome and phosphoproteome atlas of maize primary roots reveals functional gradients along the root axis. Plant Physiol. pp-00138 (2015). 10.1104/pp.15.00138 PMC4424028 25780097

[b48] Karakousis A. . Mapping and QTL analysis of the barley population Clipper × Sahara. Aust. J. Agr. Res. 54, 1137–1140 (2003).

[b49] Brown C. T., Howe A., Zhang Q., Pyrkosz A. B. & Brom T. H. A reference-free algorithm for computational normalization of shotgun sequencing data. *arXiv,* **1203.4802** [q-bio.GN] (2012).

[b50] Suzek B. E. . UniRef clusters: a comprehensive and scalable alternative for improving sequence similarity searches. Bioinformatics 31, 926–932 (2015). 25398609 10.1093/bioinformatics/btu739 PMC4375400

[b51] Langmead B. & Salzberg S. Fast gapped-read alignment with Bowtie 2. Nat. Methods 9, 357–359 (2012). 22388286 10.1038/nmeth.1923 PMC3322381

[b52] Li B. & Dewey C. N. RSEM: accurate transcript quantification from RNA-Seq data with or without a reference genome. BMC Bioinformatics 12, 323, 10.1186/1471-2105-12-323 (2011). 21816040 PMC3163565

[b53] Robinson M. D., McCarthy D. J. & Smyth G. K. edgeR: a Bioconductor package for differential expression analysis of digital gene expression data. Bioinformatics 26, 139–140 (2010). 19910308 10.1093/bioinformatics/btp616 PMC2796818

[b54] McCarthy D. J., Chen Y. & Smyth G. K. Differential expression analysis of multifactor RNA-Seq experiments with respect to biological variation. Nucleic Acids Res . 40, 4288–4297 (2012). 22287627 10.1093/nar/gks042 PMC3378882

[b55] R Development Core Team R: A language and environment for statistical computing. R Foundation for Statistical Computing, Vienna, Austria. ISBN 3-900051-07-0 (2008). Available at: http://www.R-project.org. (Accessed: 22 December 2015).

[b56] Wickham H. In ggplot2: elegant graphics for data analysis (eds Gentleman R., Parmigiani G. & Hornik K. ) (Springer, 2009).

[b57] Supek F., Bošnjak M., Škunca N. & Šmuc T. REVIGO Summarizes and Visualizes Long Lists of Gene Ontology Terms. PLoS One 6, e21800. 10.1371/journal.pone.0021800 (2011). 21789182 PMC3138752

[b58] Stanke M. & Waack S. Gene prediction with a hidden Markov model and a new intron submodel. Bioinformatics 19 (Suppl 2), ii215–ii225, 10.1093/bioinformatics/btg1080 (2003). 14534192

[b59] Amborella Genome Project. The Amborella genome and the evolution of flowering plants. Science 342, no. 6165, 10.1126/science.1241089 (2013). 24357323

[b60] Lamesch P. . The Arabidopsis Information Resource (TAIR): improved gene annotation and new tools. Nucleic Acids Res. 40, Database issue D1202–102012 (2012). 22140109 10.1093/nar/gkr1090 PMC3245047

[b61] International Brachypodium Initiative. Genome sequencing and analysis of the model grass *Brachypodium distachyon*. Nature 463, 763–768 (2010). 20148030 10.1038/nature08747

[b62] Myburg A. A. . The genome of *Eucalyptus grandis*. Nature 510, 356–362 (2014). 24919147 10.1038/nature13308

[b63] International Barley Genome Sequencing Consortium. A physical, genetic and functional sequence assembly of the barley genome. Nature , 491, 711–716 (2012). 23075845 10.1038/nature11543

[b64] Young N. D. . The Medicago genome provides insight into the evolution of rhizobial symbioses. Nature 480, 520–524 (2011). 22089132 10.1038/nature10625 PMC3272368

[b65] Ouyang S. . The TIGR Rice Genome Annotation Resource: improvements and new features. Nucleic Acids Res. 35, Database issue D883–D887 (2007). 17145706 10.1093/nar/gkl976 PMC1751532

[b66] Tuskan G. A. . The genome of black cottonwood, *Populus trichocarpa* (Torr. & Gray). Science 313, 1596–1604 (2006). 16973872 10.1126/science.1128691

[b67] Paterson A. H. . The *Sorghum bicolor* genome and the diversification of grasses. Nature 457, 551–556 (2009). 19189423 10.1038/nature07723

[b68] Schnable P. S. . The B73 maize genome: complexity, diversity, and dynamics. Science 326, 1112–1115 (2009). 19965430 10.1126/science.1178534

