# Supplementary material for: De novo transcriptome assembly and analysis of differentially expressed genes of two barley genotypes reveal root-zone-specific responses to salt exposure
Source: Sci Rep. 2016 Aug 16;6:31558. doi: 10.1038/srep31558 (PMC4985707; doi:10.1038/srep31558)

**Supplemental Figures**

# *De novo* transcriptome assembly and analysis of differentially expressed genes of two barley genotypes reveal root-zone-specific responses to salt exposure

Camilla Beate Hill^1*^, Andrew Cassin^2^, Gabriel Keeble-Gagnère^1,3^, Monika S. Doblin^2^, Antony Bacic^2^, Ute Roessner^1^

^1^School of BioSciences, The University of Melbourne, Parkville, Vic 3010, Australia, ^2^ARC Centre of Excellence in Plant Cell Walls, School of BioSciences, The University of Melbourne, Parkville, Vic 3010, Australia, ^3^School of Veterinary and Life Sciences, Murdoch University, Murdoch, WA 6150, Australia.

***Corresponding author:** Camilla B. Hill

Present address: Western Barley Genetics Alliance, Western Australian State Agricultural Biotechnology Centre, School of Veterinary and Life Sciences, Murdoch University, 90 South Street, Murdoch, WA 6150, Australia.

**Supplemental Fig S1**. Differentially expressed *Oryza sativa* high affinity potassium

transporter genes (*OsHKT*) identified in the cv. Clipper and LR Sahara *de novo* transcriptome

assemblies. Lines (colour depending on contig annotation per genotype) connect TMM normalised FPKM values. *OsHKT*, *Oryza sativa* high affinity potassium transporter gene; R, replicate; z, zone; %ID, BLASTX Percentage Identity.


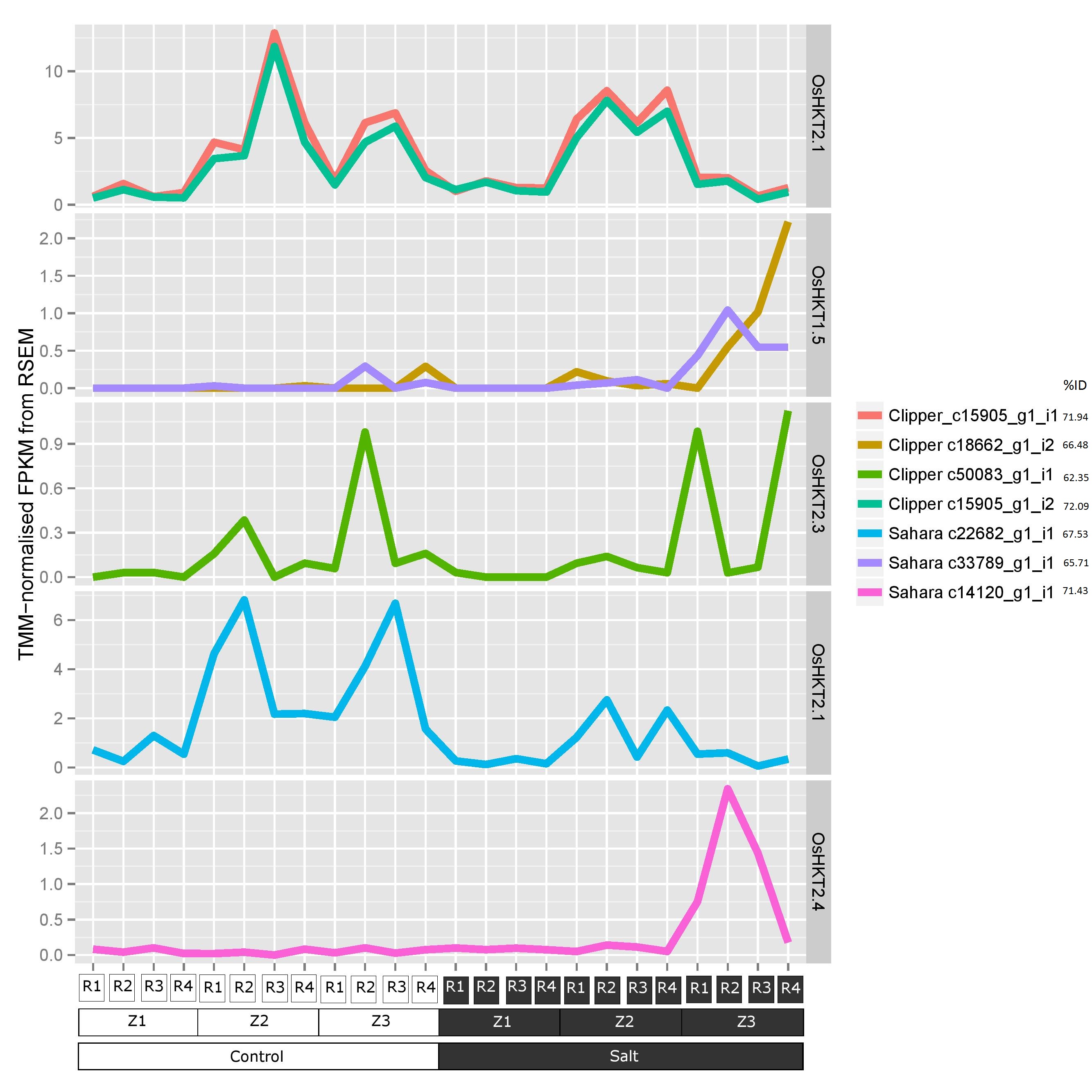


**Supplemental Fig S2**. Differentially expressed *Arabidopsis thaliana* salt overly sensitive

orthologs identified in the cv. Clipper and LR Sahara *de novo* transcriptome assemblies.

Lines (colour depending on contig annotation per genotype) connect TMM-normalised

FPKM values. *AtSOS*, Arabidopsis thaliana salt overly sensitive gene; R, replicate; z, zone; %ID, BLASTX Percentage Identity.


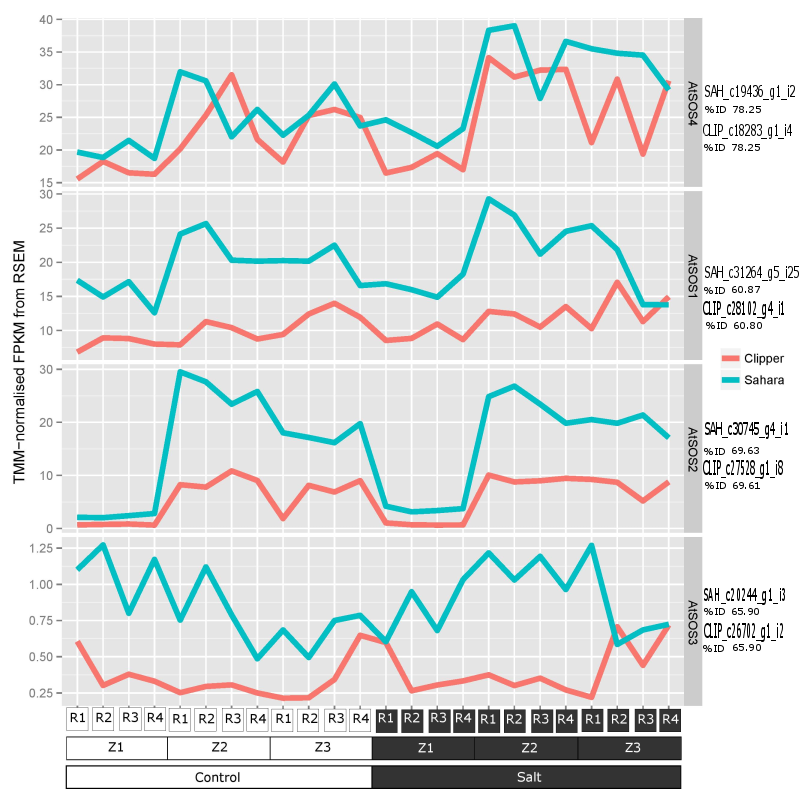


**Supplemental Fig S3**. Proportion of differentially expressed (DE, >2-fold) contigs with and

without GO annotations in cv. Clipper. The figures show multiple comparisons of different

root zones and treatments. a) DE contigs with biological process GO annotation; b) DE

contigs with molecular function GO annotation; c) DE contigs with cellular component GO

annotation .The logarithm of the fold change (log2) is displayed on the x axis, while contig

count is displayed on the y axis. Grey label: total number of contigs; colored labels: Contigs

with GO annotation (A-C); 0 mM NaCl: Control; 100: Treatment with 100 mM NaCl; Z1:

Meristematic zone; Z2: Elongation zone; Z3: Maturation zone.


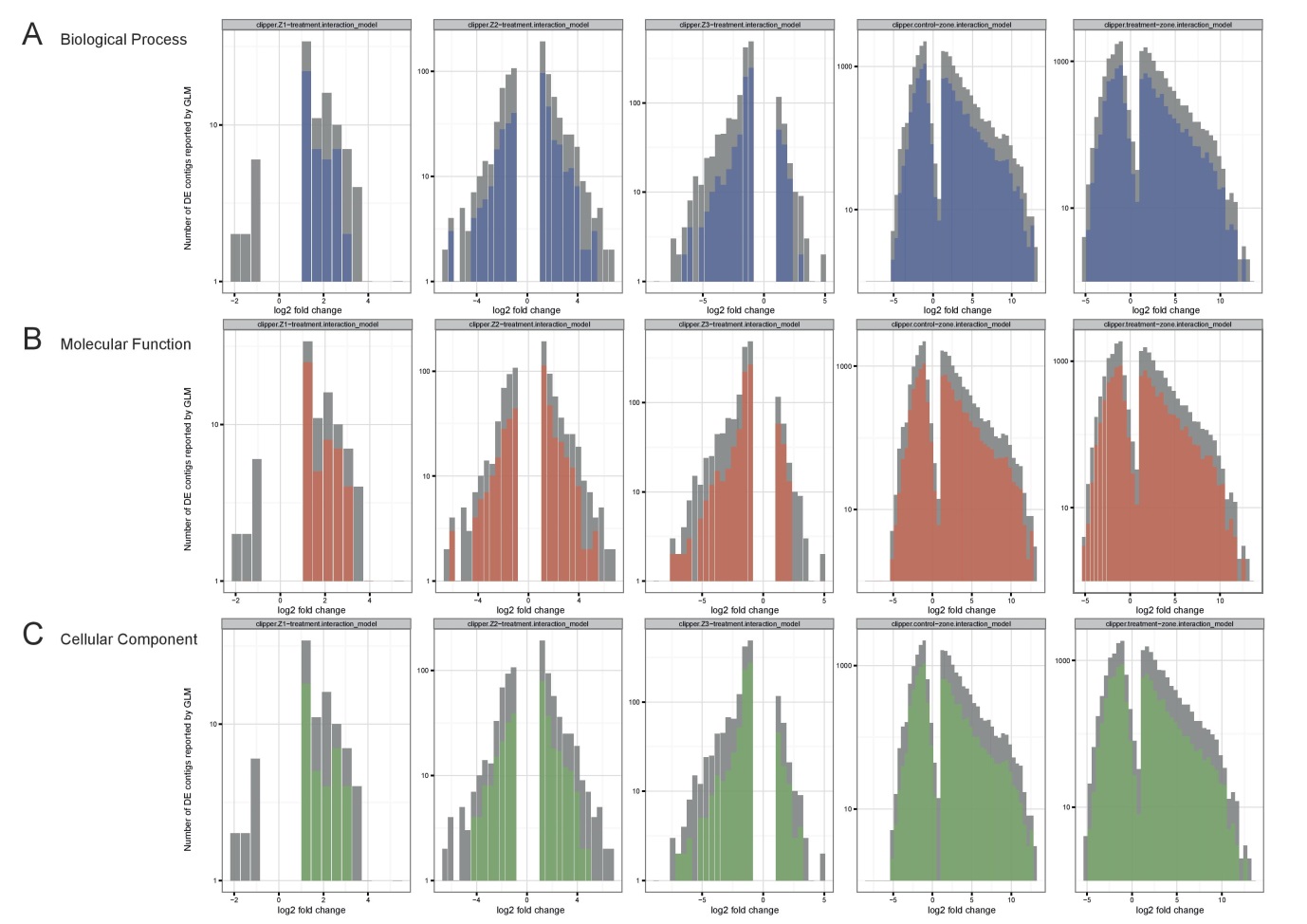


**Supplemental Fig S4**. Proportion of differentially expressed (DE, >2-fold) contigs with and

without GO annotations in LR Sahara. The figures show multiple comparisons of different

root zones and treatments. a) DE contigs with biological process GO annotation; b) DE

contigs with molecular function GO annotation; c) DE contigs with cellular component GO

annotation .The logarithm of the fold change (log2) is displayed on the x axis, while contig

count is displayed on the y axis. Grey label: total number of contigs; colored labels: Contigs

with GO annotation (A-C); 0 mM NaCl: Control; 100: Treatment with 100 mM NaCl; Z1:

Meristematic zone; Z2: Elongation zone; Z3: Maturation zone.


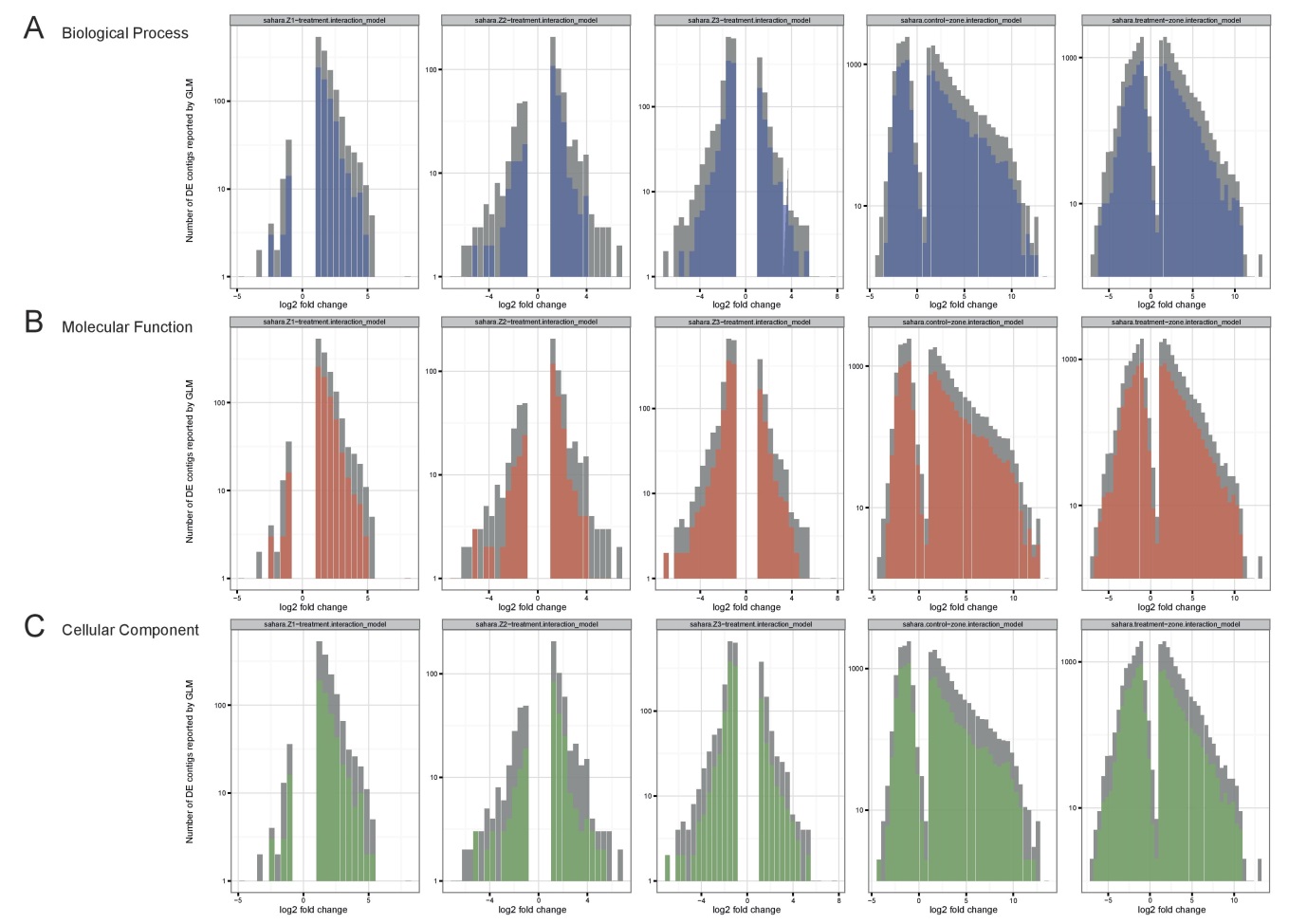

Supplement: Supplementary Information [file srep31558-s1.doc]
